# Supplementary material for: Study Protocol: Psychometric Testing of the German Vestibular Schwannoma Quality of Life Index—A Multicenter Study on Quality of Life and Patient-Centered Care in Vestibular Schwannoma
Source: Audiol Res. 2026 May 9;16(3):68. doi: 10.3390/audiolres16030068 (PMC13214468; doi:10.3390/audiolres16030068)
Supplement: Supplementary file 1 [file audiolres-16-00068-s001.zip › audiolres-4243507-supplementary.pdf]

Ziel dieses Fragebogens ist es herauszufinden, wie sich die Diagnose eines Vestibularisschwannoms (auch Akustikusneurinom genannt) oder dessen Behandlung (Operation und/oder Bestrahlung, abwartende Beobachtung bzw. „wait-and-scan“) auf Ihre Lebensqualität ausgewirkt hat. Bitte kreuzen Sie für jede Aussage die Antwortoption an, die Ihre Erfahrungen in Bezug auf Ihre Diagnose eines Vestibularisschwannoms oder dessen Behandlung am besten widerspiegelt. Wenn Sie das aufgeführte Gefühl oder die genannte Sorge nicht empfunden haben, kreuzen Sie bitte "überhaupt nicht oder betrifft mich nicht" an.

Hörprobleme:
Bitte beantworten Sie die folgenden Aussagen zu Hörproblemen. Wenn Sie ein Hörgerät tragen, berücksichtigen Sie dies bitte bei der Beantwortung der Fragen.

	überhaupt nicht oder betrifft mich nicht	ein wenig	etwas	ziemlich	sehr
1. Ich mache mir Sorgen um meine Sicherheit aufgrund meiner Hörprobleme (zum Beispiel, dass ich einen Feueralarm nicht höre, oder Schwierigkeiten habe die Richtung wahrzunehmen, aus der Fahrzeuge sich nähern).	❑	❑	❑	❑	❑
2. Ich habe Schwierigkeiten Sprache zu verstehen, wenn es Hintergrundgeräusche gibt oder mehrere Personen gleichzeitig sprechen.	❑	❑	❑	❑	❑
3. Aufgrund meiner Hörprobleme fühle ich mich ausgeschlossen, wenn ich mich in einer Gruppe von Menschen befinde.	❑	❑	❑	❑	❑
4. Ich mache mir Sorgen auch das Gehör auf meinem anderen Ohr zu verlieren (das heißt, das Ohr ohne Vestibularisschwannom).	❑	❑	❑	❑	❑
5. Meine Hörprobleme beeinträchtigen mich darin, an sozialen Freizeitaktivitäten teilzunehmen.	❑	❑	❑	❑	❑

Schwindel und Gleichgewichtsstörungen:

	überhaupt nicht oder betrifft mich nicht	ein wenig	etwas	ziemlich	sehr
6. Aufgrund meines Schwindels oder meiner Gleichgewichts-störungen habe ich Schwierigkeiten, wenn ich meinen Kopf schnell oder ruckartig bewege oder hochschaue.	❑	❑	❑	❑	❑

	überhaupt nicht oder betrifft mich nicht	ein wenig	etwas	ziemlich	sehr
7. Aufgrund meines Schwindels oder meiner Gleichgewichtss-törungen fällt es mir schwer, die Richtung während des Gehens zu ändern.	❑	❑	❑	❑	❑
8. Aufgrund meines Schwindels oder meiner Gleichgewichts-störungen habe ich Angst, dass Andere denken könnten, ich stünde unter Drogen- oder Alkoholeinfluss.	❑	❑	❑	❑	❑
9. Aufgrund meines Schwindels oder meiner Gleichgewichts-störungen, mache ich mir Sorgen ich könnte fallen oder mich verletzen.	❑	❑	❑	❑	❑
10. Aufgrund meines Schwindels oder meiner Gleichgewichts-störungen fühle ich mich nicht sicher damit Auto zu fahren.	❑	❑	❑	❑	❑
11. Mein Schwindel oder meine Gleichgewichtsstörungen be-einträchtigen mich in der Aus-führung körperlich anstrengender Aufgaben, wie zum Beispiel Sport, Tanzen oder der Gartenarbeit.	❑	❑	❑	❑	❑

**Schmerzen, Unwohlsein und Tinnitus:**					
	überhaupt nicht oder betrifft mich nicht	ein wenig	etwas	ziemlich	sehr
12. Ich leide unter einem Druckgefühl im Kopf oder Kopfschmerzen im Zusammenhang mit meiner Erkrankung.	❑	❑	❑	❑	❑
13. Aufgrund der durch meine Erkrankung hervorgerufenen Schmerzen, fühle ich mich leicht reizbar oder niedergeschlagen.	❑	❑	❑	❑	❑
14. Die durch meine Erkrankung hervorgerufenen Schmerzen schränken mich in meinen täglichen Aktivitäten ein.	❑	❑	❑	❑	❑
15. Mein Tinnitus beeinträchtigt meine Konzentrationsfähigkeit.	❑	❑	❑	❑	❑
16. Mein Tinnitus beeinträchtigt meinen Schlaf.	❑	❑	❑	❑	❑

Probleme mit den Augen oder Lähmungen im Gesichtsbereich:					
	überhaupt nicht oder betrifft mich nicht	ein wenig	etwas	ziemlich	sehr
17. Ich fühle mich aufgrund meiner Lähmungen im Gesichtsbereich unwohl.	❑	❑	❑	❑	❑
18. Meine Gesichtslähmung beeinträchtigt meine Lebens-qualität und mein tägliches Wohlbefinden.	❑	❑	❑	❑	❑
19. Es stört mich, dass mein Auge übermäßig tränt oder trocken wird.	❑	❑	❑	❑	❑
20. Mein Auge fühlt sich gereizt an oder schmerzt.	❑	❑	❑	❑	❑

Auswirkungen auf körperliches, emotionales und soziales Wohlbefinden:					
	überhaupt nicht oder betrifft mich nicht	ein wenig	etwas	ziemlich	sehr
21. Ich habe den Eindruck, dass mein allgemeiner Gesundheits-zustand schlecht ist.	❑	❑	❑	❑	❑
22. Meine Erkrankung schränkt mich in meinen täglichen Aktivitäten ein.	❑	❑	❑	❑	❑
23. Aufgrund meiner Erschöpfung habe ich Schwierigkeiten meinen üblichen Tätigkeiten nachzugehen.	❑	❑	❑	❑	❑
24. Ich habe keine Freude mehr an Dingen, die mir früher Freude bereitet haben.	❑	❑	❑	❑	❑
25. Mir gehen besorgniserregende Gedanken durch den Kopf.	❑	❑	❑	❑	❑
26. Meine Erkrankung beeinflusst meinen Blick auf das Leben negativ.	❑	❑	❑	❑	❑
27. Ich mache mir Sorgen, dass sich meine Erkrankung verschlimmert.	❑	❑	❑	❑	❑

28. Meine Erkrankung macht mich leicht reizbar oder ungeduldig.	❑	❑	❑	❑	❑
29. Meine Erkrankung belastet meine Beziehungen zu Familien-mitgliedern oder Freunden.	❑	❑	❑	❑	❑

Schwierigkeiten mit dem Denken und dem Gedächtnis:

	überhaupt nicht oder betrifft mich nicht	ein wenig	etwas	ziemlich	sehr
30. Ich habe Schwierigkeiten die richtigen Worte zu finden während ich spreche oder schreibe.	❑	❑	❑	❑	❑
31. Ich habe Schwierigkeiten mich bei beruflichen oder sozialen Veranstaltungen zu konzentrieren.	❑	❑	❑	❑	❑
32. Ich habe Schwierigkeiten mich an Dinge zu erinnern.	❑	❑	❑	❑	❑
33. Ich habe den Eindruck, dass mein Gehirn langsamer geworden ist.	❑	❑	❑	❑	❑

Zufriedenheit oder Bedauern der Behandlung:
Bitte beantworten Sie die folgenden Aussagen zu Ihren Erfahrungen mit der Behandlung (Operation und/oder Bestrahlung, abwartende Beobachtung bzw. „wait-and- scan“) Ihres Vestibularisschwannoms.

	überhaupt nicht oder betrifft mich nicht	ein wenig	etwas	ziemlich	sehr
34. Ich bin mit der Behandlung meines Vestibularisschwannoms zufrieden.	❑	❑	❑	❑	❑
35. Ich habe genügend unvoreingenommene Informationen erhalten, um eine gute Entscheidung bezüglich der Behandlung meines Vesitubularisschwannoms zu treffen.	❑	❑	❑	❑	❑
36. Bei der Behandlungsempfehlung wurde ausreichend auf meine Bedenken und Präferenzen zur Behandlung meines Vestibularisschwannoms eingegangen.	❑	❑	❑	❑	❑

37. Ich würde meinen Freund-innen, Freunden und Familienmitgliedern dieselbe Behandlung des Vestibularisschwannoms empfehlen, wenn sie in derselben Situation wären.	❑	❑	❑	❑	❑

Auswirkungen auf die berufliche Tätigkeit:
Bitte geben Sie an, ob die Einschränkungen im Zusammenhang mit der Diagnose oder der Behandlung des Vestibularisschwannoms Ihre Fähigkeit zu arbeiten, oder Ihre Arbeit wiederaufzunehmen, beeinträchtigt haben.

	Ja	Nein	Betrifft mich nicht
38. Ich musste bei der Arbeit um unterstützende Maßnahmen bitten (zum Beispiel häufigere Pausen, Live-Untertitel während digitaler Besprechungen oder Geräte bzw. Sicherheitsausrüstungen zur Unterstützung des Gleichgewichts).	❑	❑	❑
39. Ich musste mein Berufsfeld komplett ändern.	❑	❑	❑
40. Ich musste aufhören zu arbeiten und wurde auf Dauer arbeitsunfähig.	❑	❑	❑

Bitte beantworten Sie die folgenden Fragen zu Ihrer Hörsituation.
Bitte kreuzen Sie das zutreffende Kästchen an.

Linkes Ohr	Rechtes Ohr
❑ Normalhörend<br>❑ Schwerhörend<br>❑ Ertaubt	❑ Normalhörend<br>❑ Schwerhörend<br>❑ Ertaubt
Sie tragen LINKS:<br>❑ Kein Hörsystem<br>❑ ein Hörgerät<br>❑ ein implantiertes Hörsystem, und zwar ein:<br>❑ Cochlea-Implantat<br>❑ Cochlea-Implantat + integriertes Hörgerät (EAS)<br>❑ Mittelohr-Implantat<br>❑ Knochenleitungs-Implantat<br>❑ Hirnstamm-Implantat	Sie tragen RECHTS:<br>❑ Kein Hörsystem<br>❑ ein Hörgerät<br>❑ ein implantiertes Hörsystem, und zwar ein:<br>❑ Cochlea-Implantat<br>❑ Cochlea-Implantat + integriertes Hörgerät (EAS)<br>❑ Mittelohr-Implantat<br>❑ Knochenleitungs-Implantat<br>❑ Hirnstamm-Implantat

	≤ 5% (nie)	20% (sehr selten)	35% (selten)	50 (regel-mäßig)	65% (häufig)	80% (sehr häufig)	≥ 95% (immer)	nicht zutref-fend
Ermüdet Sie das Zuhören ohne Lippenlesen in einer lauten Umgebung oder größeren Gruppe?	❑	❑	❑	❑	❑	❑	❑	❑
Beeinträchtigt Ihre Hörsituation Sie im Straßenverkehr (z.B. zu Fuß, im Auto, per Fahrrad)?	❑	❑	❑	❑	❑	❑	❑	❑
Beeinträchtigt Ihre Hörsituation Ihre aktive Teilnahme beim Ausgehen oder bei sozialen Zusammenkünften (z.B. Restaurant, Bars, Feiern, Partys)?	❑	❑	❑	❑	❑	❑	❑	❑
Fällt es Ihnen schwer, einem alltäglichen Telefonat mit einer fremden Person zu folgen (z.B. mit Arzthelferin, Werkstatt)?	❑	❑	❑	❑	❑	❑	❑	❑
Fühlen Sie sich durch Ihre Hörsituation in der Kommunikation beim Einkaufen oder mit öffentlichen Stellen (z.B. bei Behördengängen, Versicherungen, Anwälten etc.) beeinträchtigt?	❑	❑	❑	❑	❑	❑	❑	❑
Beeinträchtigt Ihre Hörsituation Sie bei Ihren Aktivitäten im Alltag zu Hause (z.B. Haushalt, Pflege von Angehörigen, Beaufsichtigung von Kindern)?	❑	❑	❑	❑	❑	❑	❑	❑
Beeinträchtigt Ihre Hörsituation Sie bei der Auswahl Ihrer Freizeitaktivitäten (z.B. Ausflüge, Reisen, Sport, etc.)?	❑	❑	❑	❑	❑	❑	❑	❑
Beeinträchtigt Ihre Hörsituation den Umgang mit Ihren Mitmenschen (z.B. Freunde, Familie, Nachbarn, Sportpartner)?	❑	❑	❑	❑	❑	❑	❑	❑
Werden Sie auf Grund Ihrer Hörsituation ausgeschlossen, wenn Sie mit anderen zusammen sind?	❑	❑	❑	❑	❑	❑	❑	❑
Meiden Sie Situationen und Orte, wo Ihre Hörsituation Sie beeinträchtigen könnte?	❑	❑	❑	❑	❑	❑	❑	❑

	≤ 5% (nie)	20% (sehr selten)	35% (selten)	50% (regel-mäßig)	65% (häufig)	80% (sehr häufig)	≥ 95% (immer)	nicht zutref-fend
Beeinträchtigt Ihre Hörsituation Sie bei Ihrer Ausbildung oder Arbeit?	❑	❑	❑	❑	❑	❑	❑	❑
Fühlen Sie sich auf Grund Ihrer Hörsituation in Ihrem bekannten sozialen Umfeld unwohl?	❑	❑	❑	❑	❑	❑	❑	❑
Fühlen Sie sich auf Grund Ihrer Hörsituation im Gespräch mit Fremden unwohl?	❑	❑	❑	❑	❑	❑	❑	❑
Vermindert die Hörsituation Ihr Selbstbewusstsein oder Ihr Selbstwertgefühl?	❑	❑	❑	❑	❑	❑	❑	❑
Verspüren Sie aufgrund Ihrer Hörsituation Zukunftsängste oder Ängste über Ihre weitere Lebensplanung?	❑	❑	❑	❑	❑	❑	❑	❑
Fühlen Sie sich auf Grund Ihrer Hörsituation erschöpft oder ermüdet?	❑	❑	❑	❑	❑	❑	❑	❑
Haben Sie Schwierigkeiten, Ihre Stimme bzw. Ihre Sprechweise an verschiedene Situationen anzupassen (z.B. laut oder leise; fröhlich, traurig oder wütend)?	❑	❑	❑	❑	❑	❑	❑	❑
Hindert Ihre Hörsituation Sie daran, Ihre Wünsche und Rechte zu kommunizieren (im Beruf, in privaten Beziehungen)?	❑	❑	❑	❑	❑	❑	❑	❑
Haben Sie das Gefühl, dass Sie auf Grund Ihrer Hörsituation von anderen nicht ernst genommen werden?	❑	❑	❑	❑	❑	❑	❑	❑
Haben Sie das Gefühl, dass Sie sich auf Grund Ihrer Hörsituation im Alltag oft verhören und es zu Missverständnissen kommt?	❑	❑	❑	❑	❑	❑	❑	❑
Haben Sie das Gefühl, sich auf Grund Ihrer Hörsituation nicht gut konzentrieren zu können und sich weniger zu merken?	❑	❑	❑	❑	❑	❑	❑	❑

Mit den folgenden Fragen sollen Sie Ihren Schwindel einschätzen. Bitte beantworten Sie jede Frage mit „ja“, „manchmal“ oder „nein“. Bitte kreuzen Sie ein Kästchen für jede Frage an. Sollten Sie keinen Schwindel haben, setzen Sie bitte hier ein Kreuz und gehen zu den nächsten Fragen zu Problemen bei der Bewegung Ihres Gesichtes über:

❑ kein Schwindel

	Ja	Zeitweise	Nein
1. Haben Sie beim Nachobenschauen vermehrt Schwindelprobleme?	❑	❑	❑
2. Verstärken sich Ihre Beschwerden, wenn Sie auf einer unebenen Oberfläche laufen?	❑	❑	❑
3. Verstärken sich Ihre Beschwerden bei schnellen oder anspruchs-volleren Bewegungen, wie z.B. beim Sport, Tanzen, Hausarbeit?	❑	❑	❑
4. Verstärken schnelle Kopfbewegungen Ihre Schwindelbeschwerden?	❑	❑	❑
5. Haben Sie beim Drehen im Bett verstärkt Schwindelbeschwerden?	❑	❑	❑
6. Verstärken sich Ihre Schwindelbeschwerden beim Gehen auf Bürgersteigen an dicht befahrenen Straßen?	❑	❑	❑
7. Verstärken Nickbewegungen des Kopfes oder Überkopfarbeiten Ihre Beschwerden?	❑	❑	❑
8. Müssen Sie sich in Ihrem Berufsleben aufgrund Ihrer Schwindelbeschwerden einschränken?	❑	❑	❑
9. Haben Sie beim Lagewechsel (Aufstehen, Hinlegen) verstärkt Schwindelbeschwerden?	❑	❑	❑
10. Sind Sie aufgrund Ihrer Beschwerden im sozialen Leben eingeschränkt (Kinobesuch, Essen gehen, Tanzen gehen, Besuch von Festen)?	❑	❑	❑
11. Haben Sie beim Lesen verstärkt Probleme?	❑	❑	❑
12. Vermeiden Sie aufgrund Ihrer Beschwerden größere Höhen?	❑	❑	❑
13. Sind für Sie anstrengende Arbeiten (Haushalt oder Garten) schwer oder unmöglich?	❑	❑	❑
14. Ist es für Sie schwer, aufgrund Ihrer Beschwerden alleine außer Haus zu gehen?	❑	❑	❑
15. Ist es für Sie schwer, aufgrund Ihrer Schwindelbeschwerden im Dunkeln zu gehen?	❑	❑	❑
16. Haben Ihre Schwindelbeschwerden Einfluss auf Ihren Verantwortungsbereich im Beruf oder im Haushalt (Beschränkung auf bestimmte Tätigkeiten)?	❑	❑	❑
17. Fühlen Sie sich aufgrund Ihrer Beschwerden zeitweise frustriert?	❑	❑	❑
18. Haben Sie Angst, ohne Begleitung das Haus zu verlassen?	❑	❑	❑
19. Ist es Ihnen peinlich, wenn andere Personen Ihre Schwindelbeschwerden bzw. Unsicherheit bemerken?	❑	❑	❑
20. Haben Sie Angst davor, jemand könnte Sie für betrunken halten, wenn er Ihre Unsicherheit oder Ihr Schwanken beobachtet?	❑	❑	❑
21. Haben Sie aufgrund Ihrer Schwindelbeschwerden Konzentrationsstörungen?	❑	❑	❑
22. Haben Sie Angst, wegen Ihrer Beschwerden immer allein Zuhause sein zu müssen?	❑	❑	❑
23. Fühlen Sie sich behindert aufgrund Ihrer Schwindelbeschwerden?	❑	❑	❑
24. Haben Sie aufgrund Ihrer Beschwerden mit Ihrem Partner/Ihrer Partnerin oder der Familie bzw. Freunden schon Ärger gehabt?	❑	❑	❑
25. Fühlen Sie sich aufgrund Ihrer Beschwerden depressiv?	❑	❑	❑

Bitte kreuzen Sie die jeweils zutreffendste Antwort auf die folgenden Fragen an, die sich auf die Probleme mit der Bewegung Ihres Gesichtes beziehen.
Bitte berücksichtigen Sie dabei die Funktion Ihres Gesichtes während des vergangenen Monats:

Körperliche Funktion

1. Wie viele Schwierigkeiten hat es Ihnen bereitet, beim Essen Nahrung im Mund zu halten, Nahrung im Mund zu bewegen, oder dass sich Nahrung in der Wangentasche festsetzte?					
Meist hatte ich damit:				Meist habe ich gar nicht gegessen:	
keine Schwierigkeiten	wenig Schwierigkeiten	einige Schwierigkeiten	erhebliche Schwierigkeiten	aus gesundheitlichen Gründen	aus anderen Gründen
❑	❑	❑	❑	❑	❑
2. Wie viele Schwierigkeiten hatten Sie damit, aus einer Tasse zu trinken?					
Meist hatte ich damit:				Meist habe ich gar nicht getrunken:	
keine Schwierigkeiten	wenig Schwierigkeiten	einige Schwierigkeiten	erhebliche Schwierigkeiten	aus gesundheitlichen Gründen	aus anderen Gründen
❑	❑	❑	❑	❑	❑
3. Wie viele Schwierigkeiten hatten Sie beim Aussprechen bestimmter Laute?					
Meist hatte ich damit:				Meist habe ich gar nicht gesprochen:	
keine Schwierigkeiten	wenig Schwierigkeiten	einige Schwierigkeiten	erhebliche Schwierigkeiten	aus gesundheitlichen Gründen	aus anderen Gründen
❑	❑	❑	❑	❑	❑
4. Wie viele Schwierigkeiten hatten Sie mit einem zu stark tränenden Auge oder Augentrockenheit?					
Meist hatte ich damit:				Meist war keine Aussage zu meinem Auge möglich:	
keine Schwierigkeiten	wenig Schwierigkeiten	einige Schwierigkeiten	erhebliche Schwierigkeiten	aus gesundheitlichen Gründen	aus anderen Gründen
❑	❑	❑	❑	❑	❑
5. Wie viele Schwierigkeiten hatten Sie beim Zähneputzen und Mundspülen?					
Meist hatte ich damit:				Meist habe ich meine Zähne nicht geputzt und meinen Mund nicht gespült:	
keine Schwierigkeiten	wenig Schwierigkeiten	einige Schwierigkeiten	erhebliche Schwierigkeiten	aus gesundheitlichen Gründen	aus anderen Gründen
❑	❑	❑	❑	❑	❑

Soziale Funktion/Wohlbefinden

	Die ganze Zeit	Die meiste Zeit	Einen guten Anteil der Zeit	Einen Teil der Zeit	Eine geringe Zeit	Zu keiner Zeit
6. Wie oft haben Sie sich ruhig und friedlich gefühlt?	❑	❑	❑	❑	❑	❑
7. Wie oft haben Sie sich von ihren Mitmenschen zurückgezogen?	❑	❑	❑	❑	❑	❑
8. Wie oft waren Sie ihren Mitmenschen gegenüber gereizt?	❑	❑	❑	❑	❑	❑
9. Wie oft sind Sie zu früh aufgewacht oder nachts mehrfach aufgewacht?	❑	❑	❑	❑	❑	❑
10. Wie oft hat die Gesichtslähmung Sie davon abgehalten, auswärts essen zu gehen, einzukaufen oder an familiären oder sozialen Aktivitäten teilzunehmen?	❑	❑	❑	❑	❑	❑

Mit den folgenden Fragen sollen Sie Ihre Kopfschmerzen einschätzen. Bitte beantworten Sie jede Frage mit „ja“, „manchmal“ oder „nein“. Bitte kreuzen Sie ein Kästchen für jede Frage an. Sollten Sie keine Kopfschmerzen haben, setzen Sie bitte hier ein Kreuz und gehen zu den nächsten Fragen zu Ihrem psychischen Befinden über:

❑ keine Kopfschmerzen

	ja	manch-mal	nein
1. Aufgrund meiner Kopfschmerzen fühle ich mich beeinträchtigt.	❑	❑	❑
2. Aufgrund meiner Kopfschmerzen fühle ich mich eingeschränkt bei meinen alltägliche Aktivitäten.	❑	❑	❑
3. Keiner versteht, wie sehr sich die Kopfschmerzen auf mein Leben auswirken.	❑	❑	❑
4. Ich habe aufgrund meiner Kopfschmerzen meine Freizeitaktivitäten (z.B. Sport, Hobbys) eingeschränkt.	❑	❑	❑
5. Meine Kopfschmerzen ärgern mich.	❑	❑	❑
6. Manchmal habe ich das Gefühl, dass ich wegen meiner Kopfschmerzen die Beherrschung verlieren könnte.	❑	❑	❑
7. Durch meine Kopfschmerzen habe ich weniger soziale Kontakte.	❑	❑	❑
8. Mein Partner oder meine Familie und Freunde haben keine Vorstellung, was ich wegen meiner Kopfschmerzen durchmache.	❑	❑	❑
9. Meine Kopfschmerzen sind so schlimm, dass ich das Gefühl habe, ich könnte verrückt werden.	❑	❑	❑
10. Meine Lebenseinstellung ist durch meine Kopfschmerzen beeinflußt.	❑	❑	❑
11. Ich habe Angst auszugehen, wenn ich Kopfschmerzen bekomme.	❑	❑	❑
12. Meine Kopfschmerzen bringen mich zur Verzweiflung.	❑	❑	❑
13. Ich mach mir Sorgen, dass meine Kopfschmerzen mir im Beruf oder zu Hause Nachteile bringen könnten.	❑	❑	❑

	ja	manch-mal	nein
14. Meine Kopfschmerzen beeinträchtigen die Beziehung zu meiner Familie oder zu Freunden.	❑	❑	❑
15. Wenn ich Kopfschmerzen habe, ziehe ich mich zurück.	❑	❑	❑
16. Ich glaube, dass meine Kopfschmerzen es mir erschweren, gesteckte Ziele zu erreichen.	❑	❑	❑
17. Ich kann wegen meiner Kopfschmerzen nicht klar denken.	❑	❑	❑
18. Meine Kopfschmerzen führen zu Verspannungen (z.B. Muskelverspannungen).	❑	❑	❑
19. Ich habe an sozialen Aktivitäten keine Freude aufgrund meiner Kopfschmerzen.	❑	❑	❑
20. Ich fühle mich wegen meiner Kopfschmerzen leicht reizbar.	❑	❑	❑
21. Wegen meiner Kopfschmerzen vermeide ich Reisen.	❑	❑	❑
22. Meine Kopfschmerzen bringen mich durcheinander.	❑	❑	❑
23. Meine Kopfschmerzen frustrieren mich.	❑	❑	❑
24. Wegen meiner Kopfschmerzen fällt mir das Lesen schwer.	❑	❑	❑
25. Es fällt mir schwer, mich von meinen Kopfschmerzen abzulenken.	❑	❑	❑

	leicht	mittel	schwer
26. Wie stark sind Ihre Kopfschmerzen üblicherweise?	❑	❑	❑

27. An wieviel Tagen im Monat haben Sie Kopfschmerzen? ______________________

28. Seit wieviel Jahren leiden Sie an Kopfschmerzen? ______________________

**Wie stark fühlten Sie sich im Verlauf der letzten 2 Wochen durch die folgenden Beschwerden beeinträchtigt?**				
	überhaupt nicht	an einzelnen Tagen	an mehr als der Hälfte der Tage	beinahe jeden Tag
1. Wenig Interesse oder Freude an Ihren Tätigkeiten	❑	❑	❑	❑
2. Niedergeschlagenheit, Schwermut oder Hoffnungslosigkeit	❑	❑	❑	❑
3. Nervosität, Ängstlichkeit oder Anspannung	❑	❑	❑	❑
4. Nicht in der Lage sein, Sorgen zu stoppen oder zu kontrollieren	❑	❑	❑	❑

In diesem Fragebogen geht es um die Beurteilung Ihres Gesundheitszustandes. Der Bogen ermöglicht es, im Zeitverlauf nachzuvollziehen, wie Sie sich fühlen und wie Sie im Alltag zurechtkommen.

Bitte beantworten Sie jede Frage, indem Sie bei den Antwortmöglichkeiten die Zahl ankreuzen, die am besten auf Sie zutrifft.

	Ausge-zeichnet	Sehr gut	Gut	Weniger gut	Schlecht
1. Wie würden Sie Ihren Gesundheitszustand im Allgemeinen beschreiben?	❑	❑	❑	❑	❑

Im Folgenden sind einige Tätigkeiten beschrieben, die Sie vielleicht an einem normalen Tag ausüben. Sind Sie durch Ihren derzeitigen Gesundheitszustand bei diesen Tätigkeiten eingeschränkt? Wenn ja, wie stark?	Ja, stark eingeschränkt	Ja, etwas eingeschränkt	Nein, überhaupt nicht eingeschränkt
2. **mittelschwere Tätigkeiten,** z.B. einen Tisch verschieben, Staubsaugen, Kegeln, Golf spielen	❑	❑	❑
3. **mehrere** Treppenabsätze steigen	❑	❑	❑

Hatten Sie *in der vergangenen Woche aufgrund Ihrer **körperlichen** Gesundheit* irgendwelche Schwierigkeiten bei der Arbeit oder anderen alltäglichen Tätigkeiten im Beruf bzw. zu Hause?	Ja	Nein
4. Ich habe **weniger geschafft** als ich wollte.	❑	❑
5. Ich konnte nicht so **sorgfältig** wie üblich arbeiten.	❑	❑

Hatten Sie *in der vergangenen Woche aufgrund Ihrer **seelischen** Gesundheit* irgendwelche Schwierigkeiten bei der Arbeit oder anderen alltäglichen Tätigkeiten im Beruf bzw. zu Hause?	Ja	Nein
6. Ich habe **weniger geschafft** als ich wollte.	❑	❑
7. Ich konnte nicht so **sorgfältig** wie üblich arbeiten.	❑	❑

	Überhaupt nicht	Ein bisschen	Mäßig	Ziemlich	Sehr
8. Inwieweit haben die Schmerzen Sie in der *vergangenen Woche* bei der Ausübung Ihrer Alltagstätigkeiten zu Hause und im Beruf behindert?	❑	❑	❑	❑	❑

In diesen Fragen geht es darum, wie Sie sich fühlen und wie es Ihnen *in der vergangenen Woche* gegangen ist. (Bitte kreuzen Sie in jeder Zeile die Zahl an, die Ihrem Befinden am ehesten entspricht.)<br><br>Wie oft waren Sie *in der vergangenen Woche* …	Immer	Meistens	Ziemlich oft	Manch-mal	Selten	Nie
9. ...ruhig und gelassen?	❑	❑	❑	❑	❑	❑
10. ...voller Energie?	❑	❑	❑	❑	❑	❑
11. ...entmutigt und traurig?	❑	❑	❑	❑	❑	❑

	Immer	Meistens	Manchmal	Selten	Nie
12. Wie häufig haben Ihre körperliche Gesundheit oder seelische Probleme in der vergangenen Woche Ihre Kontakte zu anderen Menschen (Besuche bei Freunden, Verwandten usw.) beeinträchtigt?	❑	❑	❑	❑	❑

Bitte beantworten Sie die folgenden Fragen.

	trifft gar nicht zu	trifft nicht zu	teils/ teils	trifft zu	trifft völlig zu
1. Meine Hörminderung beeinträchtigt meine persönlichen Beziehungen.	❑	❑	❑	❑	❑
2. Ich habe wegen der Hörminderung Schwierigkeiten, ein Gespräch zu führen.	❑	❑	❑	❑	❑
3. Meine Konzentration wird durch Klingeln, Rauschen oder andere Geräusche im Ohr beeinträchtigt.	❑	❑	❑	❑	❑
4. Ich habe erhebliche Probleme mit Schwindel.	❑	❑	❑	❑	❑
5. Ich habe ein Unsicherheitsgefühl oder Gleichgewichtsstörungen.	❑	❑	❑	❑	❑
6. Beim Stehen oder Gehen habe ich das Gefühl, mich zu drehen oder zu fallen.	❑	❑	❑	❑	❑
7. Richtungswechsel beim Gehen bereiten mir wegen des Schwindels und der Gleichgewichts-störungen Schwierigkeiten.	❑	❑	❑	❑	❑
8. Ich habe Schwierigkeiten, mich zu Hause im Dunkeln zu bewegen.	❑	❑	❑	❑	❑
9. Ich befürchte, die Leute könnten wegen meiner Gleichgewichtsstörung denken, ich sei betrunken.	❑	❑	❑	❑	❑
10. Ich verhalte mich in Gesellschaft von Menschen anders, weil ich Schwierigkeiten habe, mein Gesicht zu bewegen.	❑	❑	❑	❑	❑
11. Ich habe ein unangenehmes Gefühl, ein Jucken oder ein starkes Tränen in einem Auge.	❑	❑	❑	❑	❑
12. Meine eingeschränkte Beweglichkeit im Gesicht hat sich auf meine Aussprache ausgewirkt.	❑	❑	❑	❑	❑
13. Aufgrund meiner Erkrankung Akustikusneurinom (Vestibularisschwannom) schaffe ich weniger, als ich möchte.	❑	❑	❑	❑	❑
14. Auf der Seite meines Akustikusneurinoms (Vestibularisschwannoms) habe ich Kopfschmerzen.	❑	❑	❑	❑	❑
15. Manchmal bekomme ich Angst, dass etwas Schlimmes passieren wird.	❑	❑	❑	❑	❑
16. Ich mache mir oft Sorgen.	❑	❑	❑	❑	❑
17. Ich fühle mich verlangsamt.	❑	❑	❑	❑	❑
18. Manchmal bin ich ängstlich und habe ein flaues Gefühl im Magen.	❑	❑	❑	❑	❑
19. Ich habe Panikattacken.	❑	❑	❑	❑	❑

	trifft gar nicht zu	trifft nicht zu	teils/ teils	trifft zu	trifft völlig zu
20. Aufgrund meiner Erkrankung Akustikusneurinom (Vestibularisschwannom) fühle ich mich oft isoliert.	❑	❑	❑	❑	❑
21. Es fällt mir manchmal schwer, mich zu konzentrieren, z.B. beim Zeitunglesen oder beim Fernsehen.	❑	❑	❑	❑	❑
22. Ich bin ungeduldiger geworden.	❑	❑	❑	❑	❑
23. Ich habe kaum noch Energie oder Antrieb.	❑	❑	❑	❑	❑
24. Ich habe Schwierigkeiten, mich an Dinge zu erinnern.	❑	❑	❑	❑	❑
25. Ich bin kerngesund.	❑	❑	❑	❑	❑
26. Ich erwarte, dass sich mein Gesundheitszustand im kommenden Jahr verschlechtern wird.	❑	❑	❑	❑	❑

In diesem Fragebogen möchten wir Sie bitten, **Angaben zu Ihren Erfahrungen mit Ihrer Nachsorge-Behandlung** zu machen. Die Befragung gibt uns die Möglichkeit, die Behandlung von Patientinnen und Patienten weiter zu verbessern. Bitte unterstützen Sie uns dabei, indem Sie den Bogen sorgfältig und vollständig ausfüllen.

Denken Sie dabei bitte an Ihren gesamten Arztbesuch während der Nachsorge: Das heißt zum Beispiel an die Anmeldung, Gespräche mit Behandelnden, Untersuchungen und Behandlungen. **Sollten Sie mehrere Ärzte/Ärztinnen zur Nachsorge aufsuchen, beantworten Sie die Frage bitte für die Fachrichtung, die Sie am häufigsten aufsuchen (z.B. Neurochirurgie).**

Die Fragen können Sie durch Ankreuzen beantworten. Es gibt keine richtigen oder falschen Antworten. Sie haben außerdem die Möglichkeit „betrifft mich nicht“ anzukreuzen, wenn Sie nicht in die jeweilige Situation kamen.

Bitte geben Sie an, wo Ihre ambulanten Nachsorgeuntersuchungen (auch Verlaufskontrollen genannt) in der Regel stattfinden.	❑ Neurochirurgie<br>❑ Hals-Nasen-Ohrenkunde	❑ Strahlentherapie<br>❑ Andere Fachrichtung

Im Folgenden geht es um den Umgang der Behandelnden mit Ihnen:

	trifft völlig zu	trifft weit-gehend zu	trifft eher zu	trifft eher nicht zu	trifft weit-gehend nicht zu	trifft über-haupt nicht zu	betrifft mich nicht
Meine Behandelnden waren einfühlsam (zum Beispiel sind sie auf meine Gefühle eingegangen, haben Verständnis gezeigt oder haben sich in meine Situation hineinversetzt).	❑	❑	❑	❑	❑	❑	❑
Die Behandelnden haben sich respektvoll und wertschätzend verhalten.	❑	❑	❑	❑	❑	❑	❑
Die Behandelnden waren engagiert, eine Lösung für meine gesundheitlichen Belange zu finden.	❑	❑	❑	❑	❑	❑	❑
Wenn ich das wollte, wurden schwierige Themen von den Behandelnden direkt und offen besprochen (zum Beispiel langfristige Folgen der Erkrankung, Lebenserwartung oder Sexualität).	❑	❑	❑	❑	❑	❑	❑

Im Folgenden geht es um das vertrauensvolle Miteinander mit Ihren Behandelnden:

	trifft völlig zu	trifft weit-gehend zu	trifft eher zu	trifft eher nicht zu	trifft weit-gehend nicht zu	trifft über-haupt nicht zu	betrifft mich nicht
Ich habe meinen Behandelnden vertraut.	❑	❑	❑	❑	❑	❑	❑
Ich hatte das Gefühl, ich konnte mich meinen Behandelnden anvertrauen (zum Beispiel bei intimen oder schwierigen Themen).	❑	❑	❑	❑	❑	❑	❑
Die Behandelnden wussten über meine Krankheitsgeschichte und meinen Gesundheitszustand Bescheid.	❑	❑	❑	❑	❑	❑	❑
Bestehende Beschwerden wurden in Folgegesprächen wieder angesprochen.	❑	❑	❑	❑	❑	❑	❑

Im Folgenden geht es darum, wie sehr auf Sie persönlich eingegangen wurde:

	trifft völlig zu	trifft weit-gehend zu	trifft eher zu	trifft eher nicht zu	trifft weit-gehend nicht zu	trifft über-haupt nicht zu	betrifft mich nicht
Meine Wünsche, Bedürfnisse und Erwartungen wurden erfragt und in der Behandlung berücksichtigt.	❑	❑	❑	❑	❑	❑	❑
Meine Behandelnden sind persönlich auf mich eingegangen und haben mich nicht als einen von vielen Menschen betrachtet.	❑	❑	❑	❑	❑	❑	❑
Es wurde gefragt und berücksichtigt, welche Ziele ich persönlich für meine Gesundheit habe.	❑	❑	❑	❑	❑	❑	❑
Es wurde gefragt und berücksichtigt, welche Möglichkeiten und Fähigkeiten ich selbst mitbringe, um meine Gesundheit zu unterstützen.	❑	❑	❑	❑	❑	❑	❑

Im Folgenden geht es um die Berücksichtigung Ihrer gesamten Lebenssituation:

	trifft völlig zu	trifft weit-gehend zu	trifft eher zu	trifft eher nicht zu	trifft weit-gehend nicht zu	trifft über-haupt nicht zu	betrifft mich nicht
Bei der Behandlung wurde meine gesamte Lebenssituation berücksichtigt (zum Beispiel Beruf, Familie und Freunde, Partnerschaft und Sexualität, Kultur und Religion, Alter oder finanzielle Verhältnisse).	❑	❑	❑	❑	❑	❑	❑
Ich wurde gefragt, wie sich meine Erkrankung auf mein Leben auswirkt.	❑	❑	❑	❑	❑	❑	❑
Meine bisherige Krankheitsgeschichte wurde erfragt und berücksichtigt.	❑	❑	❑	❑	❑	❑	❑
Ich wurde über das Zusammenspiel von körperlichen, psychischen und sozialen Faktoren informiert.	❑	❑	❑	❑	❑	❑	❑

Im Folgenden geht es um die Kommunikation mit Ihren Behandelnden:

	trifft völlig zu	trifft weit-gehend zu	trifft eher zu	trifft eher nicht zu	trifft weit-gehend nicht zu	trifft über-haupt nicht zu	betrifft mich nicht
Mir wurde genug Zeit gegeben, mein Anliegen und meine Situation zu beschreiben (zum Beispiel bisheriger Verlauf oder aktuelle Symptome).	❑	❑	❑	❑	❑	❑	❑
Die Behandelnden verwendeten Begriffe, die ich gut verstehen konnte.	❑	❑	❑	❑	❑	❑	❑
Die Behandelnden haben mich im Gespräch angesehen und mir aufmerksam zugehört.	❑	❑	❑	❑	❑	❑	❑
Die Behandelnden haben sichergestellt, dass ich richtig verstanden habe, was sie mir erklärt haben.	❑	❑	❑	❑	❑	❑	❑

Im Folgenden geht es um ergänzende Angebote zusätzlich zu Ihrer Behandlung:

	trifft völlig zu	trifft weit-gehend zu	trifft eher zu	trifft eher nicht zu	trifft weit-gehend nicht zu	trifft über-haupt nicht zu	betrifft mich nicht
Ich wurde gefragt, ob ich ergänzende Angebote nutze oder nutzen möchte (zum Beispiel Selbsthilfegruppen, Beratung, Gesundheitskurse, Alternativmedizin/ Komplementärmedizin oder spirituelle Unterstützung/ Seelsorge).	❑	❑	❑	❑	❑	❑	❑
Meine bisherige Krankheitsgeschichte wurde erfragt und berücksichtigt.	❑	❑	❑	❑	❑	❑	❑
Ich wurde über das Zusammenspiel von körperlichen, psychischen und sozialen Faktoren informiert.	❑	❑	❑	❑	❑	❑	❑
Mir wurde genug Zeit gegeben, mein Anliegen und meine Situation zu beschreiben (zum Beispiel bisheriger Verlauf oder aktuelle Symptome).	❑	❑	❑	❑	❑	❑	❑

Im Folgenden geht es um die Planung Ihrer Behandlung:

	trifft völlig zu	trifft weit-gehend zu	trifft eher zu	trifft eher nicht zu	trifft weit-gehend nicht zu	trifft über-haupt nicht zu	betrifft mich nicht
Mit mir wurde besprochen, ob Folgetermine sinnvoll sind (zum Beispiel zur Nachsorge oder Weiterbehandlung).	❑	❑	❑	❑	❑	❑	❑
Mir wurde erklärt, wie lange ich ungefähr warten muss und warum.	❑	❑	❑	❑	❑	❑	❑
Die Behandelnden haben sich genug Zeit für mich genommen.	❑	❑	❑	❑	❑	❑	❑
Die Behandlungsschritte wurden für mich schriftlich in einem Behandlungsplan festgehalten.	❑	❑	❑	❑	❑	❑	❑

Im Folgenden geht es um die Informationen, die Sie erhalten haben:

	trifft völlig zu	trifft weit-gehend zu	trifft eher zu	trifft eher nicht zu	trifft weit-gehend nicht zu	trifft über-haupt nicht zu	betrifft mich nicht
Ich habe von den Behandelnden Informationen zu meiner Erkrankung bekommen (zum Beispiel Ursachen, Symptome, Auswirkungen oder Verlauf).	❑	❑	❑	❑	❑	❑	❑
Ich wurde gefragt, was ich bereits über meine Erkrankung weiß.	❑	❑	❑	❑	❑	❑	❑
Mir wurde die Bedeutung der Untersuchungsergebnisse erklärt.	❑	❑	❑	❑	❑	❑	❑
Ich wurde gefragt was ich in Bezug auf meine Erkrankung wissen möchte.	❑	❑	❑	❑	❑	❑	❑

Herzlichen Dank für Ihre Teilnahme!
Bitte geben Sie den ausgefüllten Fragebogen in der Ambulanz ab oder senden Sie ihn im frankierten Rücksendeumschlag zurück.
